# Endoscopic Creation of an Internal Enterocolonic Fistula for a Noncommunicating Bowel Loop: A Novel Minimally Invasive Approach

**DOI:** 10.14309/crj.0000000000002059

**Published:** 2026-03-23

**Authors:** Rayhan Karimi, Andrew Dries

**Affiliations:** 1 Atrium Health, Charlotte, NC

**Keywords:** EUS-guided coloenterostomy, noncommunicating bowel loop, small bowel obstruction

## Abstract

A 62-year-old woman with long-standing short bowel syndrome on chronic total parenteral nutrition and multiple abdominal surgeries presented with acute on chronic abdominal pain and sepsis. Imaging revealed a disconnected, dilated small bowel segment in the right lower quadrant, consistent with a so-called orphaned bowel loop. Given surgical risk, she underwent a transrectal endoscopic ultrasound–guided enterocolostomy with successful creation of a coloenteric anastomosis using a 20-mm lumen-apposing metal stent. The noncommunicating bowel decompressed completely, and she achieved full symptom resolution. Follow-up endoscopy demonstrated spontaneous maturation of an internal enterocolonic fistula. This case highlights a novel, minimally invasive approach for managing noncommunicating bowel in complex surgical patients.

## INTRODUCTION

With multiple abdominal surgeries, fibrous bands known as adhesions can form, which are the most common cause of small bowel obstruction (SBO).^1^ These adhesions can lead to the formation of noncommunicating bowel loops, particularly when they result in closed loop obstructions or internal hernias.^2^ Noncommunicating bowel loops often occur in the setting of multiple abdominal surgeries. When a segment of bowel becomes excluded, it can accumulate secretions, leading to distention and increased intraluminal pressure. Furthermore, these stagnated secretions can be a source of bacterial growth leading to systemic infection. Chronic inflammation in these loops can result in fistula formation, perforation, or bowel necrosis.^3^ We present a case of woman with multiple abdominal surgeries who developed a noncommunicating bowel loop, successfully managed with an endoscopic ultrasound (EUS)-guided coloenterostomy using a lumen-apposing stent.

## CASE REPORT

A 62-year-old woman with a complex surgical and medical history presented with acute on chronic worsening abdominal pain and subjective fevers in May 2025. Her medical history was significant for short bowel syndrome on chronic total parenteral nutrition, multiple prior abdominal surgeries, and prior pulmonary embolism on apixaban.

The patient's surgical history dates to a total abdominal hysterectomy performed in 2006, which was complicated by an unrecognized small bowel injury. This missed enteric injury necessitated multiple subsequent abdominal operations, including creation of a colostomy and bypass of the right colon via a side-to-side ileotransverse colon anastomosis. In 2019, she developed complications attributable to this anastomosis and adhesive disease, including recurrent obstructive symptoms, and underwent a right hemicolectomy with extensive enterolysis. Operative findings at that time confirmed a prior ileal-to-transverse colon anastomosis with resection of approximately 50 cm of terminal ileum and the right colon proximal to the anastomosis. In December 2021, she required 2 exploratory laparotomies with small bowel resection for adhesive SBO, complicated by the development of an enterocutaneous fistula and subsequent short bowel syndrome. In November 2024, she underwent exploratory laparotomy with extensive lysis of adhesions, takedown of colocutaneous and enterocutaneous fistulae, resection of the prior ileocolonic anastomosis with redo ileocolonic anastomosis, and resection of a colorectal anastomosis with creation of a low pelvic colorectal anastomosis after splenic flexure mobilization.

Her final anatomy became very complicated, but began with the stomach, which emptied into a shortened segment of proximal small bowel, which then continued to an ileocolonic anastomosis. Distal to the ileocolonic anastomosis, the remaining colon is in continuity with a low pelvic colorectal anastomosis and rectum. A noncommunicating blind limb of small bowel is adjacent to the ileocolonic anastomosis. It was theorized that this small bowel loop had been excluded due to chronic adhesive compression. Over the next several years, she experienced recurrent admissions for SBO. In December 2023, she presented twice with worsening abdominal pain. She was managed nonoperatively during these admissions.

In May 2025, the patient again presented with acutely worsening abdominal pain and subjective fevers. She reported 5–6 bowel movements per day and continued passage of flatus, without melena, hematochezia, dysuria, or new weakness. On arrival, she was hemodynamically stable and afebrile. Physical examination revealed an alert, oriented woman in no acute distress. Cardiopulmonary examination was unremarkable. Abdominal examination showed multiple well-healed surgical scars and localized tenderness in the right lower quadrant (RLQ) without rebound or guarding.

Laboratory studies were notable for a white blood cell count of 8 ×10^3^/µL with neutrophil predominance, lactate 4.4 mmol/L, and potassium 3.3 mmol/L. She was admitted to the surgical service for further management. After fluid resuscitation and electrolyte correction, her lactate normalized. Empiric broad-spectrum coverage with piperacillin-tazobactam was initiated.

**Figure 1.**
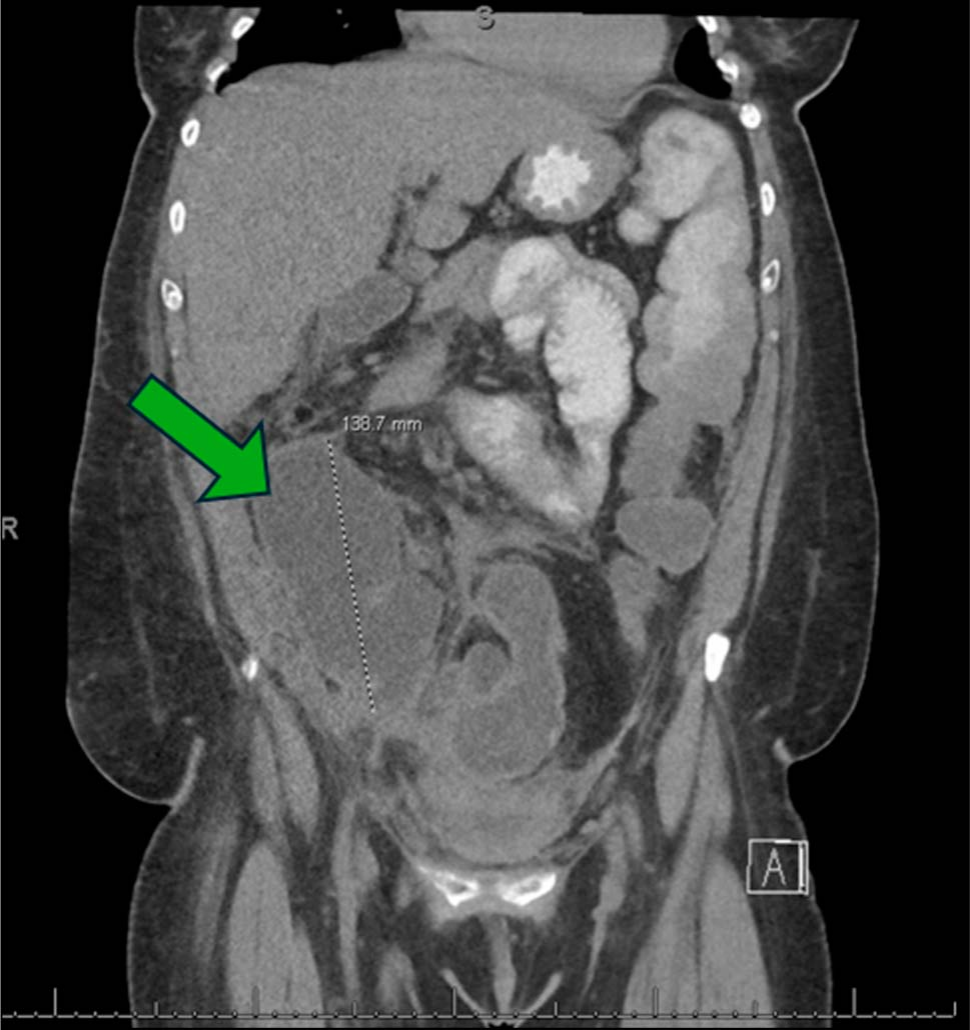
Computed tomography with oral and rectal contrast showing a complex, dilated fluid-filled tubular structure within the right lower quadrant, representing bowel, measuring 13.9 × 7.6 × 5.7 cm (green arrow).

Contrast-enhanced computed tomography (CT) of the abdomen and pelvis revealed a fluid-filled serpentine structure with rim enhancement in the RLQ, concerning for either a chronic abscess or a noncommunicating bowel loop. Despite adequate fluid resuscitation, she developed recurrent hypotension and was transferred to the surgical intensive care unit for management of septic shock. Blood cultures subsequently grew *Enterobacter* species, and her antibiotics were narrowed to cefepime and metronidazole. A repeat CT abdomen and pelvis with oral and rectal contrast demonstrated a complex, dilated, tubular structure measuring 13.9 × 7.6 × 5.7 cm in the RLQ, representing fluid-filled loops of small bowel that did not communicate with the remainder of the gastrointestinal tract—consistent with a disconnected, or “orphaned,” bowel segment, as shown in Figure 1.

Given her extensive surgical history and poor candidacy for further operative intervention, a multidisciplinary discussion was held involving surgery, interventional gastroenterology, and radiology. The consensus was to pursue an endoscopic approach to reestablish drainage and prevent ongoing sepsis from this noncommunicating loop.

**Figure 2.**
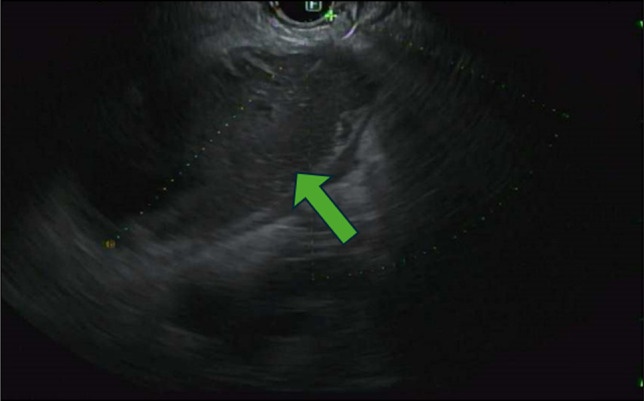
Endoscopic ultrasound from the rectum demonstrating a blind ending fluid-filled bowel loop adjacent to the rectal wall, consistent with a noncommunicating bowel segment (green arrow).

**Figure 3.**
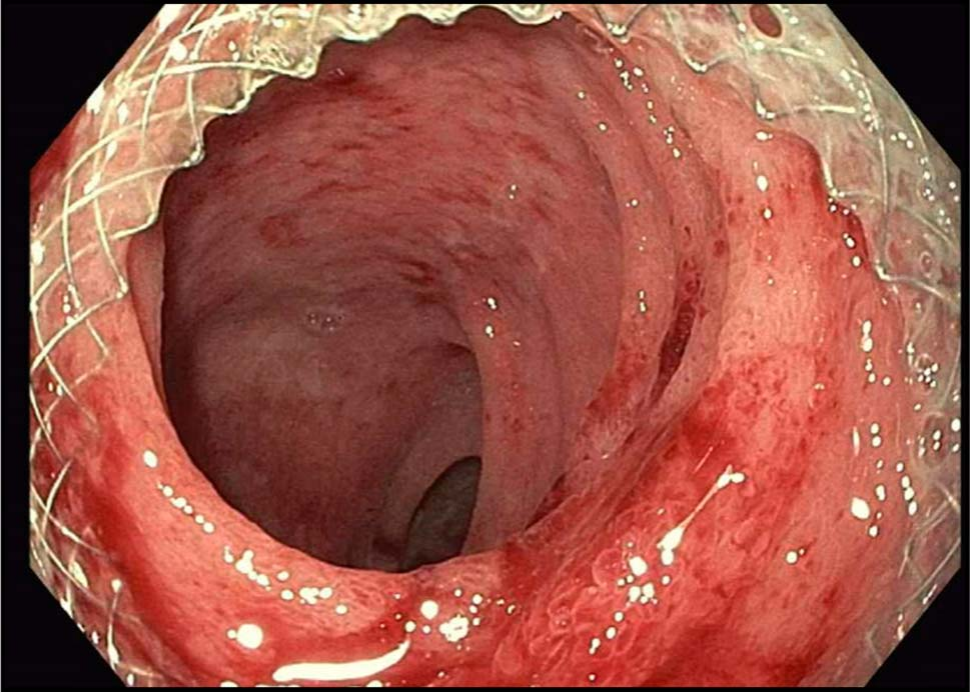
Diversion enteritis of orphaned bowel as seen through the lumen-apposing metal stent.

**Figure 4.**
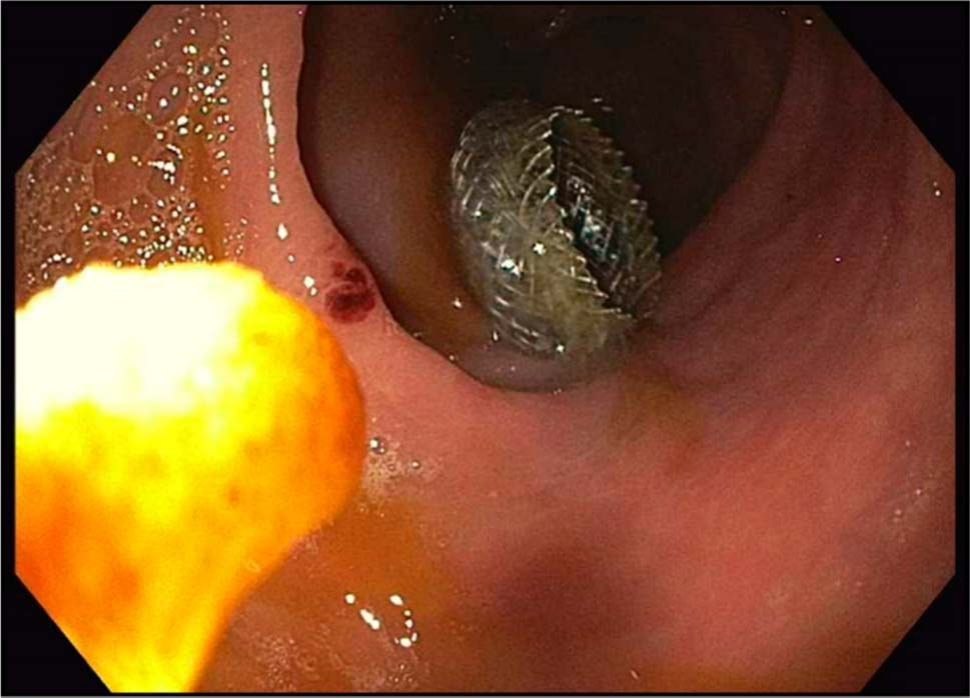
Lumen-apposing metal stent coloenterostomy fully deployed, creating communication between the colon and the noncommunicating, obstructed small bowel.

The patient underwent a lower endoscopy ultrasound. Endosonographic visualization identified a noncommunicating bowel segment adjacent to the rectosigmoid colon, as shown in Figure 2. Fine needle injection of contrast confirmed the presence of a disconnected, fluid-filled loop. Using therapeutic EUS guidance, a 20-mm lumen-apposing metal stent (LAMS) was successfully deployed to create a direct connection between the rectosigmoid colon and the noncommunicating small bowel segment—effectively performing a coloenterostomy. The stent was dilated to 15 mm under fluoroscopic guidance, allowing improved passage of the endoscope and visualization of the noncommunicating loop. Upon entrance into the excluded loop of bowel, through the stent, there was expected diversion enteritis noted, as observed in Figure 3. The open stent between the colon and noncommunicating small bowel is shown in Figure 4. Drainage of mucoid fluid was immediately observed through the stent.

**Figure 5.**
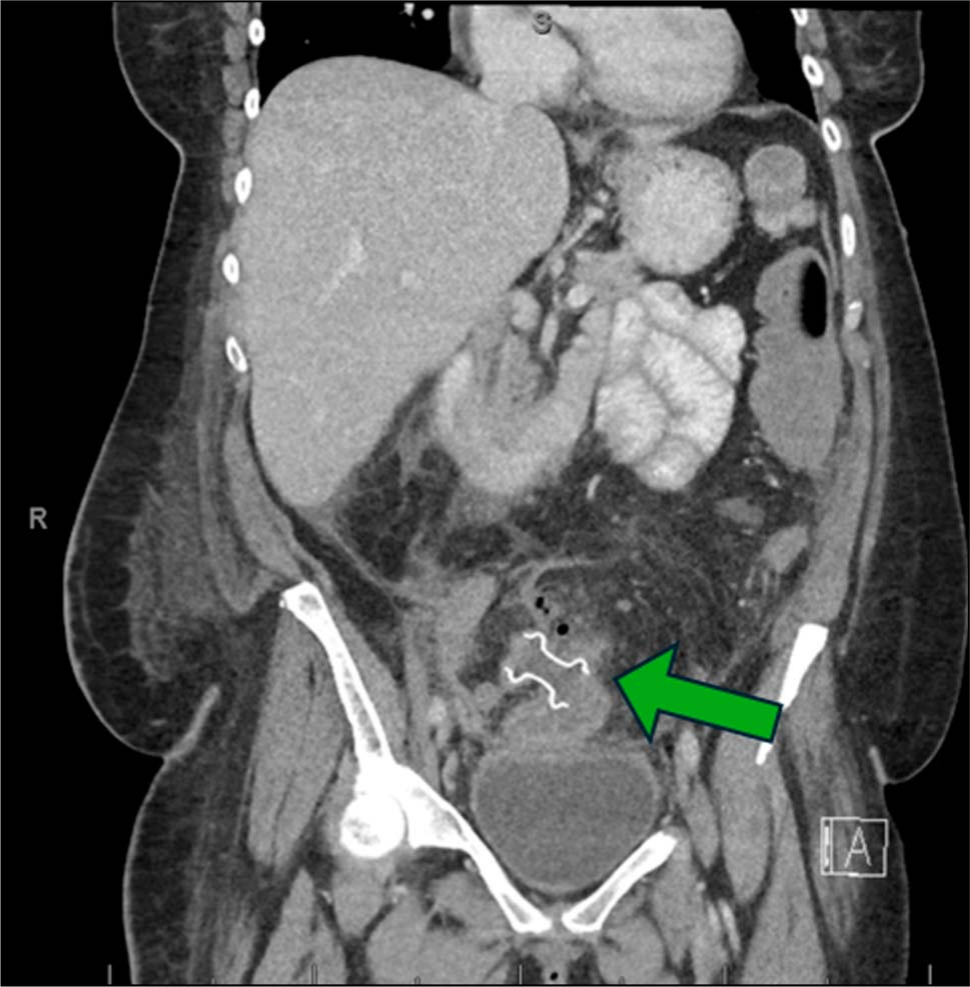
Coronal contrast-enhanced computed tomography showing interval decompression of the orphaned bowel loop after stent placement, with reduced luminal distention and no evidence of perforation or new collection (green arrow).

Following the procedure, repeat CT imaging demonstrated complete decompression of the previously dilated small bowel loops (Figure 5), confirming functional communication through the stent. Clinically, the patient experienced prompt resolution of her abdominal pain and normalization of her bowel function. She tolerated advancement to a regular diet without nausea or vomiting, and her chronic abdominal discomfort returned to baseline levels. She was discharged home on her chronic total parenteral nutrition regimen with plans for interval follow-up.

**Figure 6.**
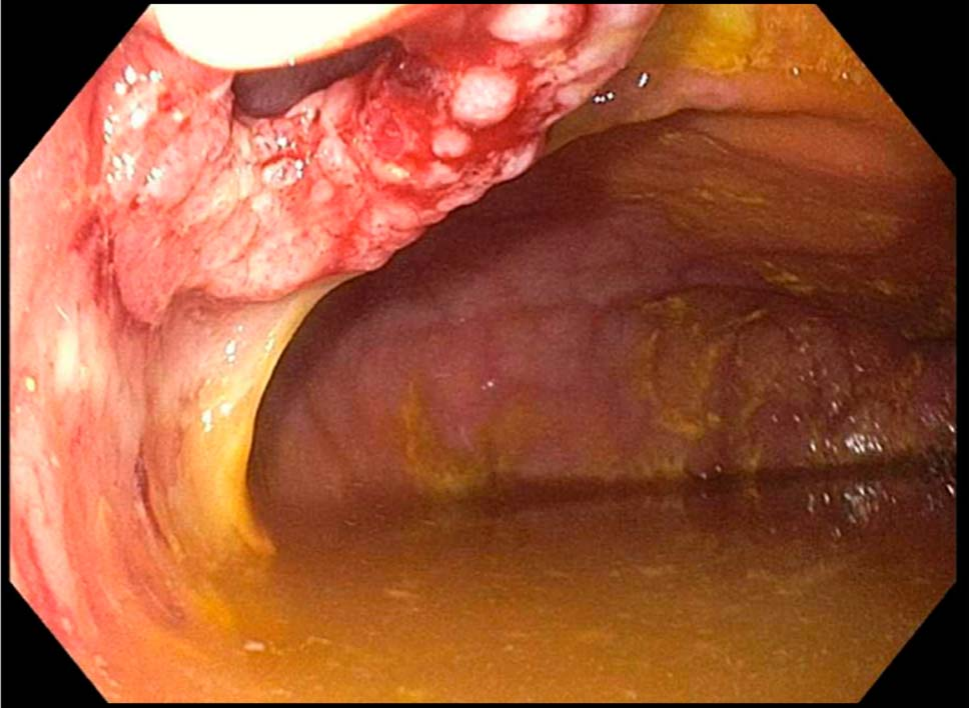
Matured internal enterocolonic fistula at prior stent site, status after lumen-apposing metal stent removal.

Two months later, she underwent elective endoscopic removal of the LAMS. Subsequent colonoscopy confirmed spontaneous maturation of an internal enterocolonic fistula at the prior stent site, maintaining drainage of the previously noncommunicating bowel segment (Figure 6). The mucosa of both the colon and the reconnected small bowel appeared healthy, with no evidence of ischemia or diversion colitis.

At her most recent follow-up several months postprocedure, the patient remained clinically stable with no further hospital admissions. She reported adequate oral intake, stable weight, and no abdominal tenderness. Follow-up imaging confirmed decompressed bowel loops and continued patency of the internal fistula.

## DISCUSSION

Patients with extensive abdominal surgical histories are predisposed to complex postoperative anatomy, including adhesions, fistulae, and formation of excluded or noncommunicating bowel loops. These loops may remain asymptomatic or lead to significant morbidity such as chronic pain, bacterial overgrowth, abscess formation, or sepsis. Although literature on noncommunicating bowel loops is limited, similar pathophysiologic processes are observed in postoperative small bowel obstructions and internal hernias, highlighting the challenges of managing such cases conservatively or surgically.^3^

In recent years, EUS-LAMS have emerged as a minimally invasive option to create internal bypasses or anastomoses, particularly in patients with malignant obstruction or surgically hostile abdomens.^4^ The use of EUS-LAMS has demonstrated to be useful in patients with surgically complicated abdomens or for palliative measures.^5–7^ A multicenter study of 8 patients undergoing EUS-guided coloenterostomy for malignant SBO demonstrated a 100% technical and 93.3% clinical success rate, with acceptable adverse events in 26.7% of patients. They displayed a median overall survival time of 65 days with a 60% 60-day survival rate. No symptom reoccurrence was identified in the first 30 days for any of the patients.^7^ This study demonstrates that EUS-coloenterostomy can be an effective alternative for palliative treatment for SBO. Another retrospective series of 26 patients studied the use of EUS-LAMS for enterocolonic bypass in malignant SBO cases that were too high risk for surgery. It showed 80% technical and 70% clinical success, underscoring the feasibility of this approach. The study also showed that the median time to oral intake was 1 day after the procedure and an average discharge to home in 6.5 days.^8^ Although EUS-coloenterostomy facilitate minimally invasive anastomoses, as with any procedure, they do carry risks including stent migration, persistent fistula, stent occlusion, ulceration, angulation, tissue overgrowth, bleeding, and rare but serious perforation or leak.^9^

In our patient, multiple prior abdominal surgeries with a “hostile abdomen” and short bowel syndrome made traditional operative management high risk. The noncommunicating bowel loop caused recurrent sepsis and pain, prompting use of an EUS-guided LAMS to establish a coloenterostomy. This intervention successfully decompressed the noncommunicating segment, resolved infection, and led to creation of a stable internal fistula without further surgery.

This case expands the use of EUS-enterostomy beyond malignant obstruction to a benign, noncommunicating loop. Although EUS-guided anastomoses are associated with favorable outcomes, careful patient selection, multidisciplinary evaluation, and close follow-up remain essential to minimize complications such as stent migration or leak.^10^ Further prospective studies are needed to define the long-term safety and durability of LAMS-mediated internal fistulas in benign, postsurgical settings.

## DISCLOSURES

Author contributions: Both authors had substantial contributions to conception of work, drafting the work, and accountability for all aspects of the work. R. Karimi is the article guarantor.

Financial disclosure: None to report.

Informed consent was obtained for this case report.

## ABBREVIATIONS:

CT: computed tomography
EUS: endoscopic ultrasound
LAMS: lumen apposing metal stent
RLQ: right lower quadrant
SBO: small bowel obstruction
